# Lower Density and Shorter Duration of Nasopharyngeal Carriage by Pneumococcal Serotype 1 (ST217) May Explain Its Increased Invasiveness over Other Serotypes

**DOI:** 10.1128/mBio.00814-20

**Published:** 2020-12-08

**Authors:** Laura Bricio-Moreno, Chrispin Chaguza, Reham Yahya, Rebecca K. Shears, Jennifer E. Cornick, Karsten Hokamp, Marie Yang, Daniel R. Neill, Neil French, Jay C. D. Hinton, Dean B. Everett, Aras Kadioglu

**Affiliations:** a Institute of Infection and Global Health, Department of Clinical Infection, Microbiology, and Immunology, University of Liverpool, Liverpool, United Kingdom; b Malawi-Liverpool-Wellcome Trust Clinical Research Programme, University of Malawi, College of Medicine, Blantyre, Malawi; c Department of Genetics, School of Genetics and Microbiology, Smurfit Institute of Genetics, Trinity College, Dublin, Ireland; d Institute of Integrative Biology, University of Liverpool, Liverpool, United Kingdom; Carnegie Mellon University

**Keywords:** pneumococcus, serotype 1, murine model, colonization, pneumonia, respiratory infection, nasopharynx, gene expression, cocolonization

## Abstract

Streptococcus pneumoniae is a frequent colonizer of the human nasopharynx and a major cause of life-threating invasive infections such as pneumonia, meningitis and sepsis. Over 1 million people die every year due to invasive pneumococcal disease (IPD), mainly in developing countries. Serotype 1 is a common cause of IPD; however, unlike other serotypes, it is rarely found in the carrier state in the nasopharynx, which is often considered a prerequisite for disease. The aim of this study was to understand this dichotomy. We used murine models of carriage and IPD to characterize the pathogenesis of African serotype 1 (sequence type 217) pneumococcal strains obtained from the Queen Elizabeth Central Hospital in Blantyre, Malawi. We found that ST217 pneumococcal strains were highly virulent in a mouse model of invasive pneumonia, but in contrast to the generally accepted assumption, can also successfully establish nasopharyngeal carriage. Interestingly, we found that cocolonizing serotypes may proliferate in the presence of serotype 1, suggesting that acquisition of serotype 1 carriage could increase the risk of developing IPD by other serotypes. RNA sequencing analysis confirmed that key virulence genes associated with inflammation and tissue invasiveness were upregulated in serotype 1. These data reveal important new insights into serotype 1 pathogenesis, with implications for carriage potential and risk of invasive disease through interactions with other cocolonizing serotypes, an often-overlooked factor in transmission and disease progression.

## Abstract (precis)

The pneumococcus causes serious diseases such as pneumonia, sepsis, and meningitis and is a major cause of morbidity and mortality worldwide. Serotype 1 accounts for the majority of invasive pneumococcal disease cases in sub-Saharan Africa but is rarely found during nasopharyngeal carriage.

## INTRODUCTION

Streptococcus pneumoniae is a commensal of the nasopharynx, responsible for local infections such as otitis media, sinusitis, and bronchitis, as well as life-threatening invasive diseases such as pneumonia, meningitis, and sepsis (1). The pneumococcus predominantly affects children, the elderly, and the immunocompromised, placing an enormous burden on the health care systems of developing countries, where there remains a high incidence of pneumococcal diseases (1, 2).

There are more than 90 different pneumococcal serotypes, which are determined by the composition and structure of the polysaccharide capsule (3, 4). The pneumococcal capsule is an important virulence factor that provides protection against phagocytosis by preventing complement deposition and by reducing the interaction with antibodies (5, 6).

Pneumococcal carriage rates vary substantially depending on age and geographical location, ranging from 30 to 90% in children under the age of 5 years to <10% in adults (6–9). Although most serotypes are commonly found in the nasopharynx of healthy adults, serotype 1 is rarely detected during nasopharyngeal carriage, but it remains a common cause of invasive pneumococcal disease (IPD) worldwide (10, 11). In sub-Saharan Africa, serotype 1 accounts for approximately 20% of all cases of IPD, and approximately 60% of those are sequence type (ST) 217 or ST217 single-locus variants (12–16). The 13-valent conjugate vaccine (PCV13), which includes serotype 1, was introduced in Malawi in November 2011, but it is less effective against this serotype (14, 15). While the vaccine provides protection against serotype 1 disease in infancy, carriage and transmission among older children and adults is suspected as the cause of serotype 1 disease persisting in the population (16). Even though serotype 1 is a common cause of invasive disease in sub-Saharan Africa, nasopharyngeal carriage rates with this serotype are around 2%, as opposed to commonly carried serotypes such as 6A, 23F, and 19F, with colonization rates of approximately 20% (10, 12). It is not clear why serotype 1 has such low carriage potential but such high levels of invasiveness.

The spread of serotype 1 has been shown to be linked to person-to-person contact in closed communities (17), suggesting that carriage events may be short-lived. This is an interesting prospect, since carriage is known to be an essential stage for disease and horizontal gene transfer; hence, reduced carriage rates would suggest that serotype 1 should encounter fewer opportunities for genetic recombination than other serotypes, leading to a reduced genetic diversity among serotype 1 isolates (10). However, published data from our group show levels of recombination in serotype 1 in line with those reported in serotypes commonly detected during nasopharyngeal carriage, suggesting that serotype 1 may actually carry for periods long enough to allow extensive recombination (12).

Phylogeographic analysis based on whole-genome sequencing has shown that serotype 1 isolates cluster into four distinct lineages, with each lineage predominantly associated with a single continent. Serotype 1 isolates from Africa (lineage B) are genomically distinct from those from Europe, a finding that is reflected in the pathogenic differences between sequence types (12). A number of studies have attempted to explain the unusual epidemiological patterns of serotype 1 infection; however, these have focused on European serotype 1 isolates (18, 19). The aim of the present study was to understand the mechanisms of infection of African ST217 pneumococcal serotype 1 during nasopharyngeal carriage and invasive pneumococcal disease. Since differential gene expression patterns between bacteremia and meningitis isolates have been previously documented (20), one ST217 isolate obtained from the blood of a sepsis patient and one ST217 isolate obtained from the cerebrospinal fluid (CSF) of a meningitis patient at the Queen Elizabeth Central Hospital in Blantyre (Malawi) were used in this study.

In contrast to the generally accepted view of low carriage potential, our findings demonstrate that serotype 1 carries readily in the nasopharynx but at a lower density and for a shorter duration than is typical for other pneumococcal serotypes. In line with this, comparative analysis of the expression of key virulence genes showed reduced expression of genes associated with nasopharyngeal carriage (e.g., neuraminidases) and increased expression of genes associated with invasive disease (e.g., pneumolysin). Interestingly, we have also shown that serotype 1 may enhance the carriage density of other cocolonizing serotypes, suggesting that its prevalence could influence and increase the incidence of non-serotype 1 pneumococcal disease as well.

## RESULTS

### Serotype 1 ST217 can colonize the nasopharynx for at least 14 days.

**FIG 1:**
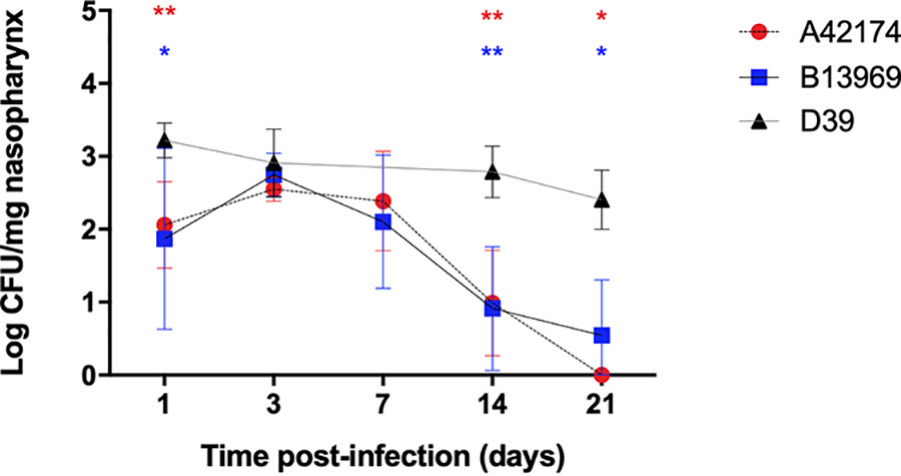
Nasopharyngeal carriage of serotype 1 ST217 bacteremia (A42174) and meningitis (B13969) isolates and serotype 2 (strain D39). Geometric means ± standard deviations (SD) of bacterial numbers in the nasopharynges of mice inoculated with 1 × 10^5^ CFU of serotype 1 bacteremia (red circles) or meningitis (blue squares) isolates or serotype 2 strain D39 (black triangles) during a 21-day period were determined. There were five mice per group per time point. *, *P* < 0.05; **, *P* < 0.01 (analyzed by one-way ANOVA and *post hoc* Dunnett’s method).

A mouse model of stable, long-term pneumococcal carriage (21) was used to determine whether serotype 1 ST217 strains could successfully colonize the nasopharynx. As previously described, D39 was able to stably colonize the nasopharynx of MF1 mice at approximately 10^3^ CFU/mg of tissue for at least 14 days (21). However, although serotype 1 ST217 strains isolated from bacteremia (A42174) and meningitis (B13969) cases were able to establish nasopharyngeal carriage over the first 7 days, the density at which they colonized the nasopharynx of MF1 mice declined over the course of the experiment (Fig. 1). Initially, serotype 1 colonized at a significantly lower CFU than D39, at ∼10^2^ CFU/mg, and then proliferated over the next 3 days before reaching a period of stabilization between days 3 and 7 postinfection. This was followed by a gradual reduction in bacterial numbers over the next 2 weeks, leading to bacterial clearance by day 21, in contrast to D39, which successfully maintained a stable level of carriage over the same 21-day period. No bacteria were recovered from the lungs or blood at any time point for any strain. None of the mice showed any signs of illness. As no differences were observed between the two serotype 1 ST217 isolates, the bacteremia (A42174) isolate was used in all subsequent *in vivo* studies.

### Nasopharyngeal carriage with serotype 1 ST217 induces proinflammatory cytokine production.

**FIG 2:**
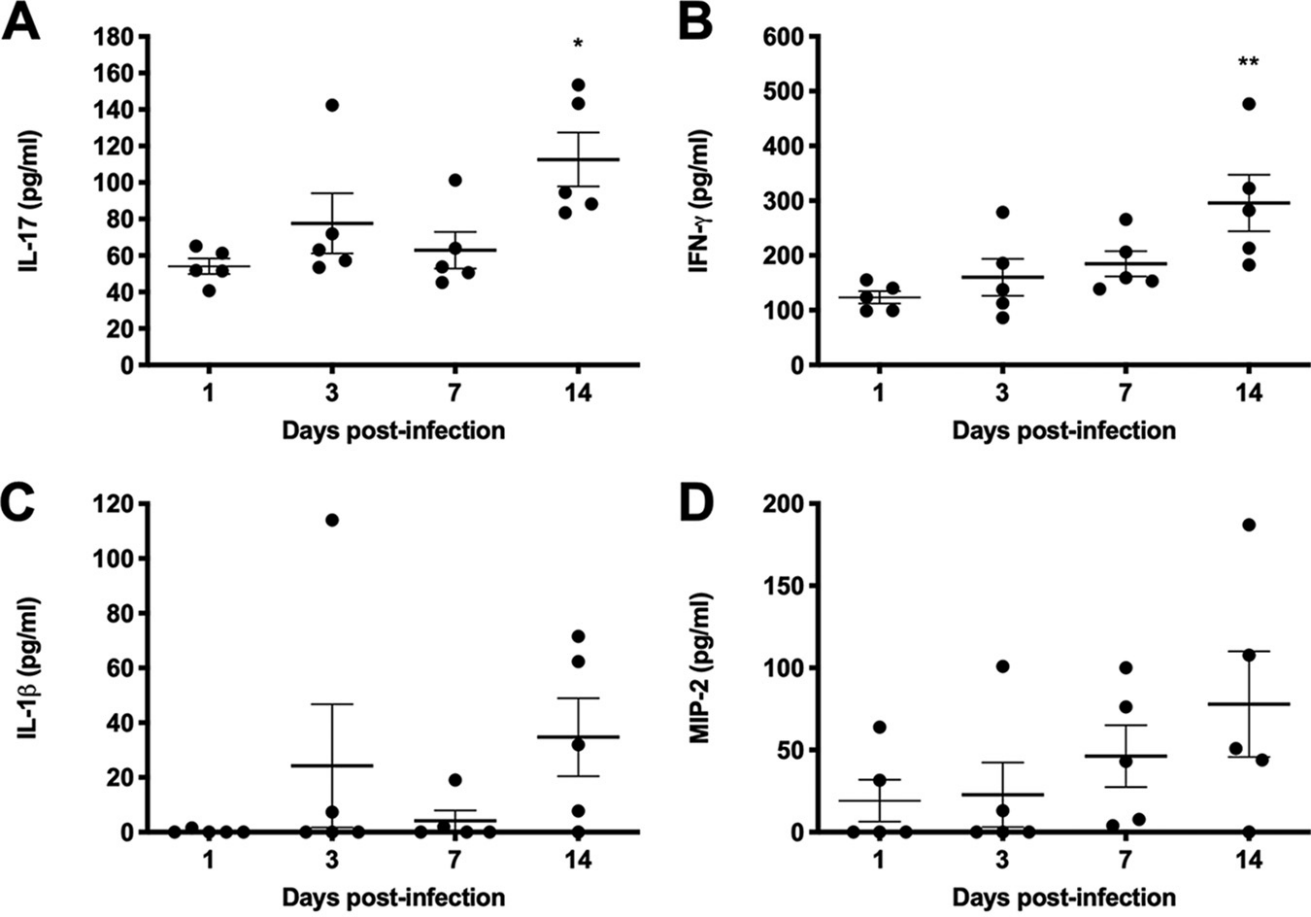
Cytokine levels in the nasopharynx during 14-day colonization with serotype 1 ST217. Means ± the SD of the nasopharyngeal concentrations of IL-17 (A), IFN-γ (B), IL-1β (C), and MIP-2 (D) were determined in mice carrying serotype 1 ST217 at days 1, 3, 7, and 14 postinfection. *, *P* < 0.05; **, *P* < 0.01 (analyzed by two-way ANOVA and *post hoc* Dunnett’s method).

During nasopharyngeal carriage with serotype 1 ST217, significant increases in nasopharyngeal interleukin-17 (IL-17) and gamma interferon (IFN-γ) levels were observed over the 14-day period of carriage (Fig. 2A and B). In line with this and at an equivalent time point, the bacterial CFU of ST217 in the nasopharynx had been significantly reduced to a level of almost complete clearance (Fig. 1A), suggesting a potential role for this cytokine in bacterial clearance from the nasopharynx. Although not statistically significant, the levels of the proinflammatory cytokines IL-1β and macrophage inflammatory protein 2 (MIP-2) also increased over time in the nasopharynx of serotype 1 ST217 infected mice (Fig. 2C and D). The levels of IL-10 and transforming growth factor β1 (TGF-β1) did not vary throughout the 14-day time course of carriage (data not shown), supporting the observation of a proinflammatory response to serotype 1 carriage.

### Nasopharyngeal carriage with serotype 1 ST217 is only partially protective against future carriage episodes.

**FIG 3:**
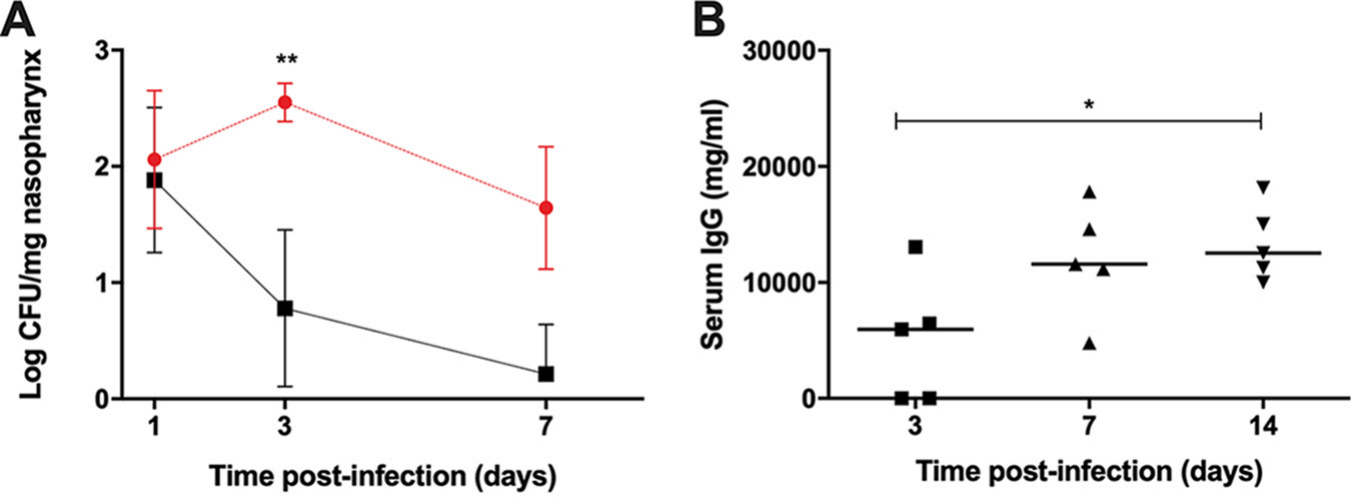
Serotype 1 ST217 CFU and serum IgG levels during initial and recarriage events. (A) CFU of serotype 1 ST217 in the nasopharynges of naive mice (red circles) and mice reinfected with the same strain 21 days after the initial infection (black squares). **, *P* < 0.01 (analyzed by two-way ANOVA with the Sidak multiple-comparison method). (B) Total serum IgG in mice carrying serotype 1 ST217 at 3, 7, and 14 days postinfection after one colonization event. Both infections were performed by intranasally inoculating 1 × 10^5^ CFU. Data are geometric means ± the SD of three to five mice per time point for each condition. *, *P* < 0.05 (analyzed by one-way ANOVA with the Tukey multiple-comparison test).

A reinfection experiment for nasopharyngeal carriage was used to assess whether the shorter duration of nasopharyngeal carriage in serotype 1 ST217-infected mice was sufficient to induce protective immunity against future colonization episodes. Mice were infected with the standard carriage dose of 10^5^ bacteria in 10 μl of phosphate-buffered saline (PBS). Twenty-one days later, when mice had cleared the bacteria from the original infection, they were rechallenged with the same dose of the same serotype 1 ST217 isolate. Bacterial numbers in the nasopharynx were determined 1, 3, and 7 days after the second challenge (Fig. 3A). Compared to naive mice exposed to the primary carriage event, the rechallenged mice had reduced pneumococcal CFU, with 60% of rechallenged mice having cleared their CFU by 7 days after reinfection compared to 100% colonization rates in naive mice from primary exposure alone. Mouse sera from the primary carriage event showed increasing total IgG serum levels by day 14 (Fig. 3B). Thus, low-density, short-term carriage with serotype 1 affords only partial protection against recolonization, suggesting that carriage episodes with serotype 1 may not be completely immunizing events.

### Nasopharyngeal cocolonization with other serotypes reduces density and duration of serotype 1 ST217 carriage.

**FIG 4:**
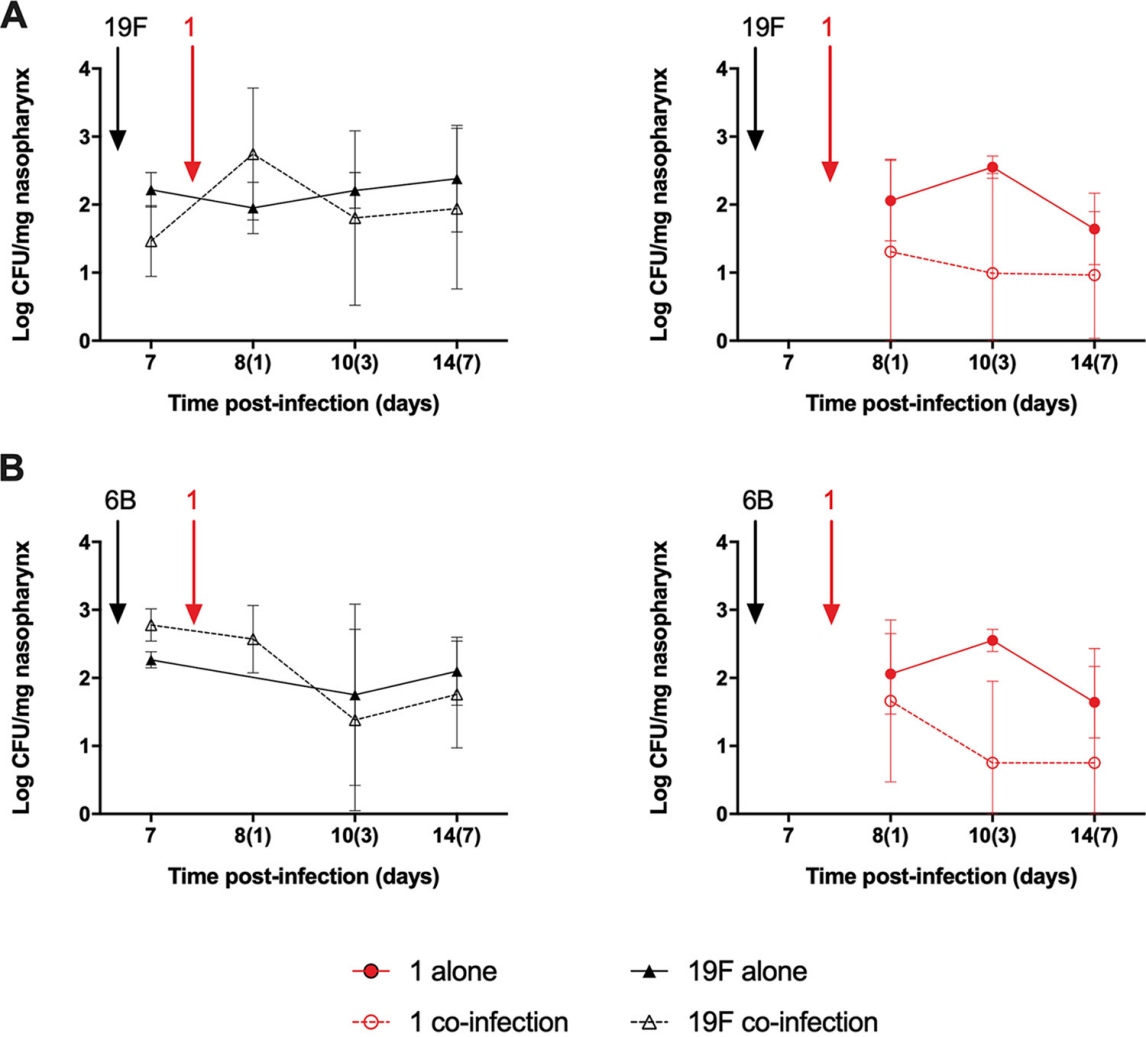
Cocolonization model with a 19F or 6B strain and serotype 1 ST217. Geometric means ± the SD of the bacterial numbers from serotype 1 and 19F (A) or 6B (B) in the nasopharynges of five mice/group/time point infected initially with serotypes 19F or 6B, followed 7 days later by coinfection with serotype 1. *, *P* < 0.05 (analyzed by two-way ANOVA).

A cocolonization model was used in which mice were initially colonized with either a serotype 19F or 6B strain and then reinfected 7 days later with serotype 1 ST217 strain (Fig. 4). Serotypes 19F and 6B were chosen due to their prevalence and high carriage rates in Malawi (22). When serotype 1 ST217 was introduced into mice already pre-colonized with serotype 19F or 6B (Fig. 4A and B), there was a reduction in the density of cocolonized serotype 1 over the next 7 days compared to equivalent time points in mice colonized only with serotype 1. Interestingly, within 24 h of cocolonization with serotype 1, a temporary increase in 19F CFU was observed compared to mice colonized only with serotype 19F (Fig. 4A). These findings suggest that cocolonization with other serotypes is detrimental to serotype 1 carriage density and that the introduction of serotype 1 into the nasopharyngeal niches of precolonized mice may cause an increase in the bacterial density of carriage of the first colonizing serotype.

### Differential expression of pneumococcal genes between serotypes 1 ST217 and 2 D39.

The gene expression profile of bacterial pathogens during *in vitro* growth can reflect their pathogenic lifestyle; invasive variants of S. pneumoniae have been reported to express higher levels of invasion-associated genes than noninvasive variants (23–26). Therefore, we compared the *in vitro* expression pattern of genes known to be involved in (i) the metabolism of carbohydrates, (ii) intraspecies competition, and (iii) virulence in serotype 1 ST217 strain and the serotype 2 (D39) strain. Three stages of growth were analyzed: early exponential phase (EEP), mid-exponential phase (MEP), and late exponential phase (LEP). The analysis is focused on the exponential phase of growth of the pneumococcus as this bacterium undergoes autolysis during stationary phase (27). We found that up to 61% of genes were differentially expressed between D39 and ST217, with 28 to 41% of these genes being downregulated in serotype 1 (see Table S1 in the supplemental material).

10.1128/mBio.00814-20.3 TABLE S1 Number of differentially expressed orthologous genes in the serotype 1 ST217 strain and the serotype 2 D39 strain. The numbers of genes differentially expressed in the serotype 1 ST217 strain compared to serotype 2 D39 during the three exponential growth phases—early exponential phase (EEP), mid-exponential phase (MEP), and late exponential phase (LEP)—are shown. A total of 1,607 orthologous genes were included in this analysis. The data were filtered to only take into account TPM (transcripts per million) values of >10 and fold changes of >2. Download Table S1, XLSX file, 0.01 MB. Copyright © 2020 Bricio-Moreno et al. 2020 Bricio-Moreno et al. This content is distributed under the terms of the Creative Commons Attribution 4.0 International license.

**FIG 5:**
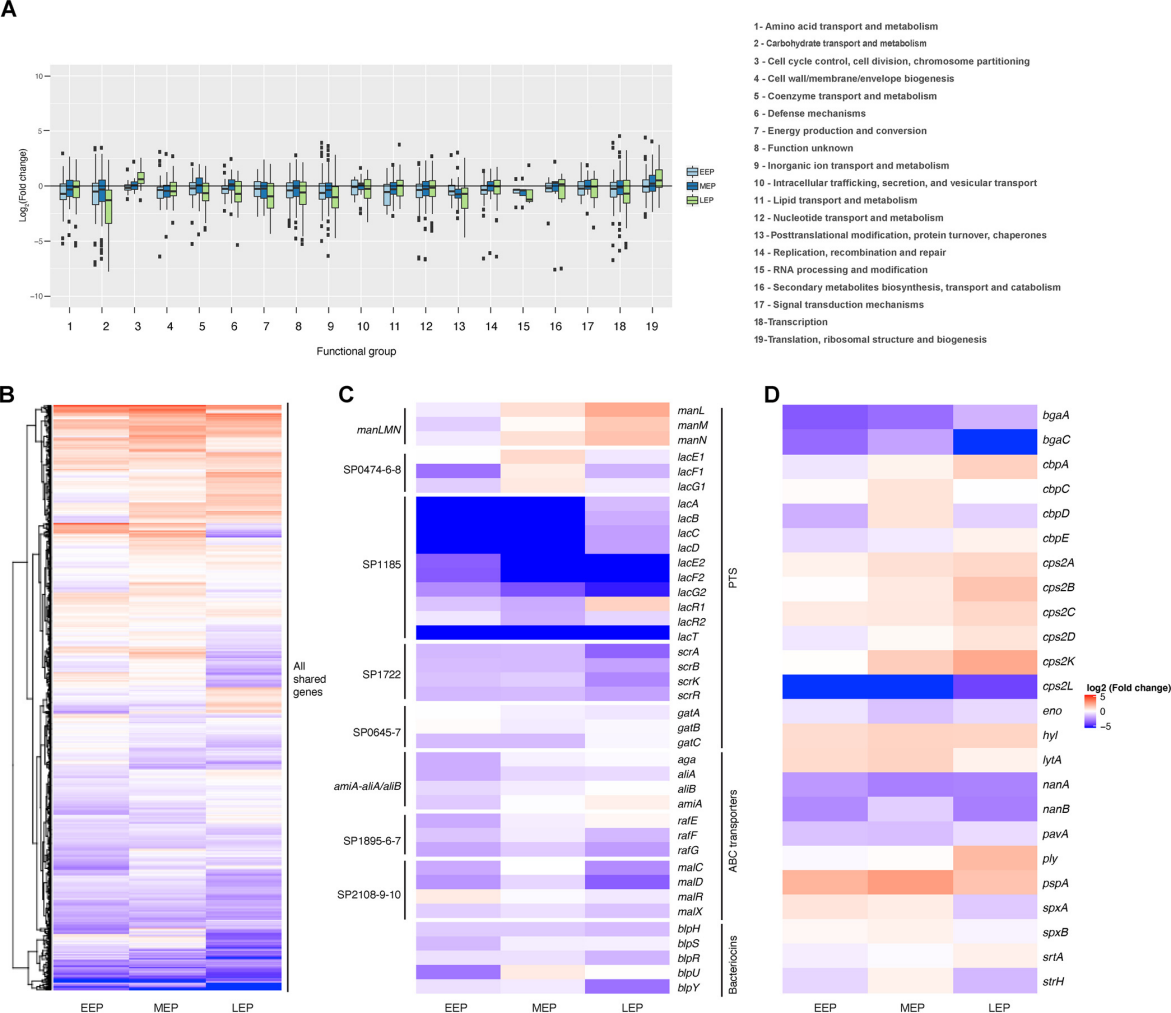
Differential gene expression between the serotype 1 ST217 and serotype 2 D39 strains. (A) Differential gene expression of the orthologous genes in serotype 1 ST217 and serotype 2 D39 strains by functional annotation during the three exponential phases: early exponential phase (EEP), mid-exponential phase (MEP), and late exponential phase (LEP). (B to D) *In vitro* pairwise comparisons of all genes (B), operons involved in pneumococcal metabolism and competition (C), and important pneumococcal virulence factors (D) showing shared genes differentially expressed in serotype 1 ST217 and serotype 2 D39 strains during the three exponential phases. The figure shows the logarithm to base 2 of the ratio of the transcripts per kilobase million (TPM) for each gene in the serotype 1 ST217 strain relative to the serotype 2 D39 strain. Only genes with available functional annotation information are shown in the figure. An *in vitro* pairwise comparison for the full list of genes shared between the two strains can be found in Fig. S1 in the supplemental material. Additional information on the functional codes and groups for the genes is provided in Table S2 in the supplemental material.

We analyzed the differential gene expression of the orthologous genes in serotype 1 ST217 and serotype 2 D39 strains by functional annotation during the three stages of growth (Fig. 5A), and we observed that those genes involved in carbohydrate transportation and metabolism were highly downregulated in serotype 1 compared to D39. We then focused on comparing the gene expression of five phosphotransferase systems (PTS) and four ATP-binding cassette (ABC) transporters (Fig. 5C) since some of these carbohydrate transport systems have previously been associated with either colonization or invasive disease (28). The operons associated with colonization are the two lactose type PTS SP0474-6-8 and SP1185 (28), the SP1722 glucose type PTS (28), the SP0645-7 galactitol type PTS system (29), the Ami-AliA/AliB oligopeptide permease ABC transporter (30, 31), and the SP2108-9-10 ABC transporter (same regulon as *malA*, *malP*, *malQ*, and *malR*) (32–34). These metabolic operons were mainly downregulated or non-differentially expressed in serotype 1. In contrast, the operons associated with virulence are the *manLMN* PTS system (28, 35) and the ABC transporter SP1895-6-7 (31, 36, 37), which were upregulated or mildly downregulated, respectively, in serotype 1 during the mid-exponential and late exponential phases of growth.

10.1128/mBio.00814-20.1 FIG S1 *In vitro* pairwise comparison of all shared genes differentially expressed in serotype 1 ST217 and serotype 2 D39 strains during the three exponential phases. The figure shows the logarithm to base 2 of the ratio of the transcripts per kilobase million for each gene in the serotype 1 ST217 strain relative to the serotype 2 D39 strain. Only genes with available functional annotation information are shown. Download FIG S1, PDF file, 0.1 MB. Copyright © 2020 Bricio-Moreno et al. 2020 Bricio-Moreno et al. This content is distributed under the terms of the Creative Commons Attribution 4.0 International license.

10.1128/mBio.00814-20.4 TABLE S2 Additional information for all the genes from the serotype 1 ST217 and serotype 2 D39 strains. The information includes the TPM (transcripts per million) values and the functional codes and groups for all the genes expressed in the serotype 1 ST217 and serotype 2 D39 strains. Download Table S2, XLSX file, 0.5 MB. Copyright © 2020 Bricio-Moreno et al. 2020 Bricio-Moreno et al. This content is distributed under the terms of the Creative Commons Attribution 4.0 International license.

We also studied the differential gene expression of the *blp* locus, which encodes pneumococcal bacteriocins and has been previously shown to drive growth inhibition in strains from different serotypes (38). We found that genes from this locus were either non-differentially expressed or downregulated in serotype 1, suggesting that serotype 1 might be at a disadvantage during cocolonization compared to other serotypes, such as D39 (Fig. 5C). Our cocolonization data from Fig. 4 would support this theory.

Previous studies have identified *in vivo* niche-specific pathways involved in differential carbon source utilization that are specific for the lung (proline from glutamate) or the nasopharynx (UMO from glutamine, the glucose-glycogen-glucose cycle, and arginine from glutamine) (39). We studied the differential gene expression of these pathways in serotype 1 and serotype 2; however, these data were inconclusive (see Fig. S2 in the supplemental material). We found that the genes required uniquely in the lung are upregulated in serotype 1 compared to serotype 2; however, the genes required uniquely in the nasopharynx were found to be both upregulated and downregulated in serotype 1 compared to serotype 2.

10.1128/mBio.00814-20.2 FIG S2 *In vitro* pairwise comparison of genes from *in vivo* niche-specific pathways differentially expressed in serotype 1 ST217 and serotype 2 D39 strains during the three exponential phases. The proline from the glutamate pathway is specific to the lung, while the UMO from glutamine, the glucose-glycogen-glucose cycle, and the arginine from glutamine pathways are specific to the nasopharynx. The figure shows the logarithm to base 2 of the ratio of the transcripts per kilobase million for each gene in the serotype 1 ST217 strain relative to the serotype 2 D39 strain. Only genes with available functional annotation information are shown. Download FIG S2, TIF file, 0.5 MB. Copyright © 2020 Bricio-Moreno et al. 2020 Bricio-Moreno et al. This content is distributed under the terms of the Creative Commons Attribution 4.0 International license.

In addition to operons, we also studied the differential gene expression of key pneumococcal virulence factors (Fig. 5D), some of which were well-defined virulence factors involved in adherence and colonization of the airway epithelium, such as the surface exoglycosidases (BgaA, BgaC, NanA, NanB, and StrH), choline-binding proteins (CbpA, CbpC, CbpD, CbpE, PspA, and LytA), enolase (Eno), and sortase A (SrtA) (6, 40–48). We found that the expression of these virulence factors was, throughout the different phases, either down-regulated, non-differentially expressed or mildly up-regulated in serotype 1 ST217, with the exception of CbpA, PspA, and LytA. Interestingly, we found that these virulence factors were upregulated in serotype 1; however, these virulence factors have also previously been shown to be important for invasion. CbpA has been shown to be involved in translocation, which is essentially involved in tissue invasiveness; PspA prevents binding of C3 onto pneumococcal surface, therefore reducing pneumococcal killing; and LytA helps digests the cell wall, which results in the release of the pneumococcal toxin Ply (6, 45). Other virulence factors have previously been shown to be directly involved in invasiveness, such as pneumolysin (*ply*), hyaluronidase lyase (*hyl*), and capsule genes (*cps2A*, *cps2B*, *cps2C*, *cps2D*, *cps2K*, and *cps2L*) (4, 49–52). These genes were upregulated in serotype 1, with the exception of *cps2L* (Fig. 5D); however, the pneumococcal adherence and virulence factor A (PavA) (53–55) was downregulated.

The observed upregulation of genes involved in pneumococcal translocation and virulence, in addition to the downregulation of genes involved in pneumococcal adherence and carriage, may offer a plausible reason for why serotype 1 ST217 is highly invasive but rarely found in human carriage.

### Blood survival.

**FIG 6:**
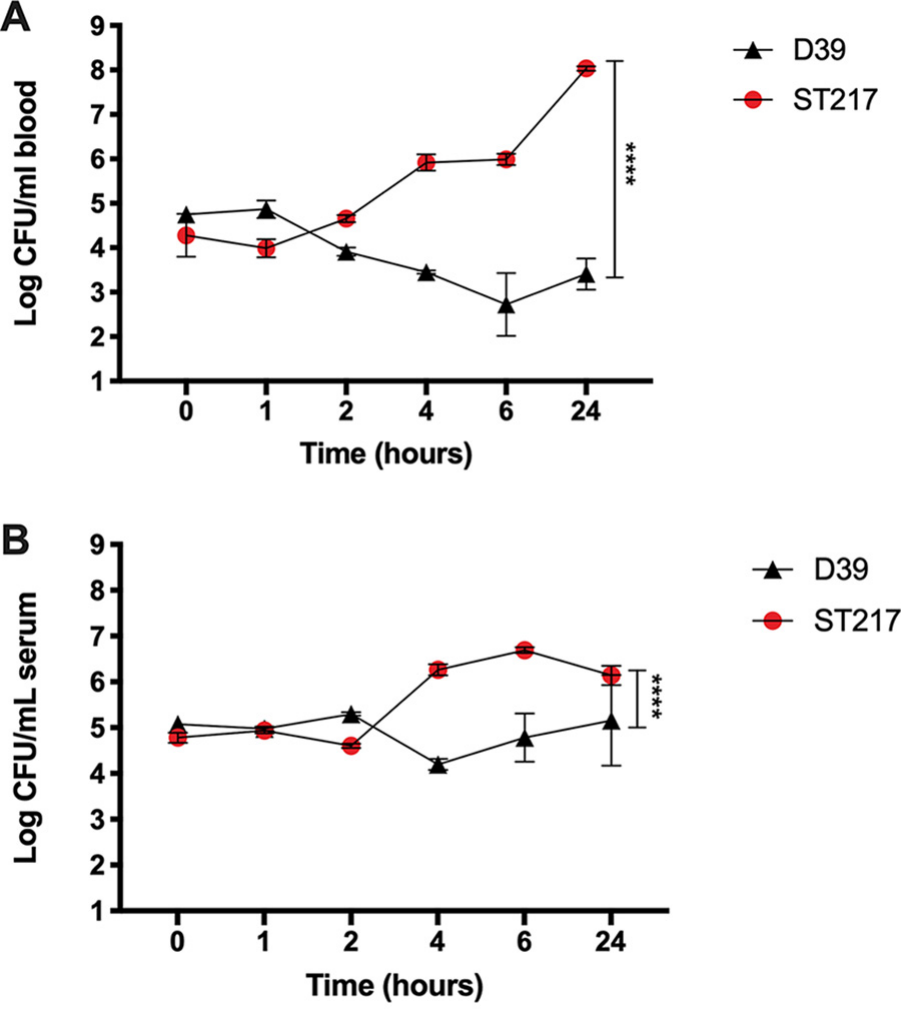
Survival assay in blood and serum with serotype 1 ST217 (A42174) and serotype 2 (D39). CFU of D39 and ST217 after incubation of 10^5^ CFU in 300 μl of whole human blood (A) or serum (B) for 1, 2, 4, 6, and 24 h. The data shown are geometric means ± the SD of two individual experiments performed in triplicate. Both serotype and time point were significant sources of variation in two-way ANOVA (*P* < 0.0001 for both).

In order to further investigate the increased virulence of serotype 1 compared to serotype 2, survival of both serotypes in human blood and serum was determined over a 24-h period. After incubation with whole human blood, it was observed that the CFU of serotype 2 D39 steadily declined up to 2 logs during the first 4 h of incubation, only to remain stable at 10^3^ CFU/ml between 4 and 24 h (Fig. 6A). However, the CFU of serotype 1 ST217 strain steadily increased during the first 6 h and then rapidly increased 100-fold between 6 and 24 h of incubation. Both serotype and time point were significant sources of variation in two-way analysis of variance (ANOVA). When the survival of both serotypes was tested in serum, we observed a similar pattern, where serotype 1 rapidly increased between 2 and 4 h postinfection; however, it remained stable for the next 20 h (Fig. 6B). In contrast, there was a reduction in serotype 2 CFU between 2 and 4 h postinfection, only to remain relatively constant for the next 20 h. Both serotype and time point were significant sources of variation in two-way ANOVA. These results confirm that serotype 1 is better adapted to survive in human blood and serum than serotype 2. However, serotype 1 seems to be better adapted to surviving in whole blood than in serum, which could be partly explained by increased resistance to neutrophils killing.

**FIG 7:**
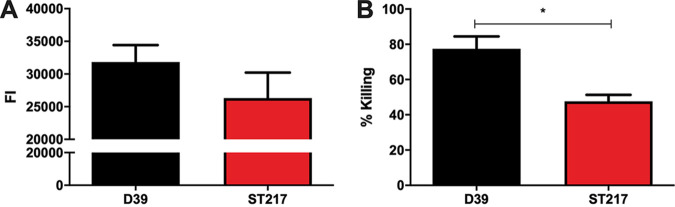
Complement deposition and resistance to opsonophagocytosis of serotype 1 ST217 strain and serotype 2 D39 strain. (A) Human C3 deposition represented by the fluorescence intensity (FI) on the serotype 2 strain D39, and serotype 1 strain. (B) Percent killing of the serotype-2 strain D39 and serotype-1 strain by HL-60 cells after a 30 min of incubation with 1:3 intravenous IgG as the opsonin. Percent killing was calculated by comparing the samples to the control without HL-60 cells. The sata shown are means ± the standard errors of the mean of three individual experiments performed in triplicate. *, *P* < 0.05 (analyzed using a Student *t* test).

We next explored two other components of systemic immune defense, assessing the levels of C3 deposition and associated phagocytosis of the two strains. We found a 17% reduction in the levels of C3 deposition on the surface of serotype 1 ST217 strain compared to D39 (Fig. 7A). Although this difference was not statistically significant (*P* = 0.1810), in line with higher levels of C3 deposition on D39, opsonophagocytic killing assays with differentiated HL60 neutrophils showed that there was a significantly higher percentage of opsonophagocytic killing of D39 compared to serotype 1 ST217 strain (*P* < 0.05) (Fig. 7B). Decreased complement deposition and reduced phagocytic killing may play an important part in increased serotype 1 survival in blood.

## DISCUSSION

Streptococcus pneumoniae serotype 1 ST217 is a major cause of invasive pneumococcal disease worldwide, with disease incidence as high as 30% in developing countries. However, unlike other serotypes, serotype 1 is rarely isolated from nasopharyngeal carriage (56). This is highly unusual, since carriage is considered to be a prerequisite for invasive disease (57). To further explore this question, we investigated the carriage potential of serotype 1 *in vivo* and compared it to serotype 2 strain D39.

We found that although the potential to cause invasive disease in mice was similar for both serotype 1 and 2 strains (data not shown) the pattern of carriage was different. D39 has previously been shown to reproducibly colonize the nasopharynx of mice for a minimum of 28 days (21, 53, 58); in the present study, we show that, serotype 1 ST217 strains were also able to colonize the nasopharynx of mice, but for a shorter period of time and at a lower density than D39. As such, serotype 1 can be recovered during nasopharyngeal carriage but is cleared 14 days postinfection, despite nearly equivalent bacterial loads during the first 7 days of carriage to D39 (Fig. 1). It has previously been shown that an increase in regulatory T cells and their immunomodulatory cytokines IL-10 and TGF-β1 is required for noninflammatory maintenance and longer duration of stable carriage by the pneumococcus (58). However, during nasopharyngeal carriage with serotype 1 ST217, the levels of IL-10 and TGF-β1 remained low throughout the experiment. The lack of induction of such immunomodulatory cytokines might lead to increased levels of proinflammatory cytokines, such as IL-17, IFN-γ, IL-1β, and MIP-2 (59), resulting in bacterial clearance. Indeed, serotype 1 ST217 was cleared from the nasopharyngeal niche after day 14 (Fig. 1), a finding in line with increasing levels of these cytokines (Fig. 2) and increases in total IgG levels in serum (Fig. 3B). In data not shown, we observed a nonsignificant slight increase in the number of neutrophils on day 1 during serotype 1 carriage, which corresponds with the peak in bacterial density. In addition, we observed a nonsignificant increase in the number of CD4 T cells, which correlates with the increase in IL-17 on day 14. We hypothesize that an initial increase in neutrophils might kick start the clearance of serotype 1, which is finalized by the action of CD4^+^ Th17 cells.

To gain some insight into potential drivers of these differences, we studied the differential expression of operons and genes linked to increase prevalence of pneumococcal nasopharyngeal carriage. The pneumococcus relies exclusively on carbohydrates as a carbon source; however, the availability of simple carbohydrates is limited in the airway; hence, the pneumococcus acquires carbon through modification and import of complex glycans (28). To this end, a number of carbohydrate import systems, which belong mainly to the ATP-binding cassette (ABC) superfamily or to the phosphotransferase systems (PTS), are utilized by the pneumococcus (32), with more than 30 carbohydrates uptaken *in vitro* (27). We found that genes involved in carbohydrate transport and metabolism were downregulated in serotype 1 ST217 compared to serotype 2 (Fig. 5A). It is believed that gain and loss of metabolic pathways is highly influenced by the host niche (32), with each transporter providing an advantage to either colonization, transmission or pathogenesis (28). The operons previously linked to nasopharyngeal carriage are the two lactose type PTS SP0474-6-8 and SP1185 (28), the SP1722 glucose type PTS (28), the SP0645-7 galactitol type PTS system (29), the Ami-AliA/AliB oligopeptide permease ABC transporter (30, 31), and the SP2108-9-10 ABC transporter (same regulon as *malC*, *malD*, *malR*, and *malX*) (32–34). The pneumococcal virulence factors previously linked to nasopharyngeal carriage are the surface exoglycosidases (BgaA, BgaC, NanA, NanB, and StrH), choline binding proteins (CbpA, CbpC, CbpD, CbpE, PspA, and LytA), enolase (Eno), and the sortase A (SrtA) (6, 40–48). We found that these genes and operons, which are linked to nasopharyngeal carriage, were downregulated or non-differentially expressed in serotype 1 ST217 compared to serotype 2 (Fig. 5), suggesting that serotype 1 has a reduced ability to adhere and therefore a reduced ability to sustain nasopharyngeal colonization. However, although these genes have been linked to nasopharyngeal carriage, some of them have been shown to be important during both nasopharyngeal carriage and invasion (6, 45). Those genes were *cbpA*, *lytA*, and *pspA*, which were upregulated in ST217 compared to serotype 2.

In contrast, we also studied the following operons and virulence factors linked to increased invasiveness: the *manLMN* PTS system (28, 35), the ABC transporter SP1895-6-7 (31, 36, 37, 60), and the virulence factors pneumolysin (Ply), the pneumococcal adherence and virulence factor A (PavA), hyaluronidase lyase (Hyl) and the orthologous capsule genes (*cps2A*, *cps2B*, *cps2C*, *cps2D*, *cps2K*, and *cps2L*) (4, 49–55). We found that these genes, operons, and virulence factors involved in invasiveness were upregulated in serotype 1 compared to serotype 2, with the exception of SP1895-6-7, which showed similar levels of expression in both serotypes, and PavA, which is linked to both adherence and invasiveness (53–55).

These observations suggest that serotype 1 might have an increased ability to translocate, become invasive, and evade the host immune responses. We studied the ability of serotype 1 to evade host immune responses and to survive in human blood and serum, and we found that serotype 1 has a significantly greater ability to survive in human blood and serum and is substantially better at resisting complement deposition and phagocytic killing than serotype 2 (Fig. 6 and 7), making it better adapted to resist host clearance mechanisms during invasive disease. However, although the pneumococcal capsule has an important effect on the ability of the pneumococcus to resist host defense mechanisms, the genetic background and differential gene expression can also play an important role in pneumococcal pathogenicity (61).

During cocarriage experiments where serotype 1 was introduced into the nasopharynx of mice precolonized by 19F or 6B, we found that the ability of serotype 1 to colonize was diminished in the presence of a previous colonizer compared to single colonization events (Fig. 4). Interestingly, when the first colonizer was serotype 19F, the bacterial density of 19F was increased after serotype 1 was introduced as the cocolonizing strain (Fig. 4A). However, the mechanisms behind this remain unknown.

Bacteriocins are antibacterial proteins that cause intraspecies competition within the host during nasopharyngeal colonization, allowing some strains to predominate over others (38, 62). The reduced ability of serotype 1 to colonize the nasopharynx of mice already colonized by other pneumococcal serotypes and the downregulation of bacteriocin genes (Fig. 5D) suggest that serotype 1 is even less capable of colonizing the nasopharynx when other serotypes are already present. This is an intriguing observation since serotype 1 mainly affects young adults, who have low rates of pneumococcal colonization (12, 63, 64), possibly explaining why serotype 1 is best adapted to inhabit niches less occupied by other pneumococcal serotypes.

Serotype 1 is one of the serotypes included in the pneumococcal conjugate vaccine (PCV13). Although PCV13 has been proven to be successful at reducing carriage rates by other vaccine covered serotypes, the vaccine has shown reduced efficiency against serotype 1 due to its complex transmission and its force of infection (65, 66). Our study shows that in the presence of other colonizing serotypes, the colonizing ability of serotype 1 can be reduced. This may suggest that vaccine induced reductions in nasopharyngeal carriage by other serotypes may open a niche for colonization by serotype 1, which could lead to increased carriage rates of serotype 1 and therefore increased levels of invasive disease caused by this serotype. In addition, serotype 1 could change the pathogenicity of other serotypes already colonizing the nasopharynx by increasing their carriage density. Therefore, increased levels of serotype 1 might lead to increased mortality caused not only by serotype 1 itself but also by other serotypes directly affected by increased prevalence of serotype 1 carriage. These observations emphasize the importance of developing an efficient vaccine against serotype 1, which would lead to a reduction of invasive pneumococcal disease by this serotype and possibly other highly prevalent serotypes.

## MATERIALS AND METHODS

### Ethics statement.

This study was performed under Animals (Scientific Procedures) Act 1986 guidelines. The protocols were approved by both the UK Home Office and the University of Liverpool Animal Welfare and Ethics Committee, and all experiments were performed at the University of Liverpool under Home Office project license 40/3602. The experiments were carried out in line with the 3R requirements, using the minimum number of animals, minimizing suffering, and using *in vitro* alternatives where available.

### Mouse strains.

Outbred, female MF1 mice (Charles River, UK) were used to model pneumococcal nasopharyngeal carriage and invasive pneumococcal disease. Mice were between 6 and 10 weeks old and were left to acclimatize for 1 week before use.

### Bacterial isolates.

Two ST217 S. pneumoniae serotype 1 strains were used in this study: strain B13969 was isolated from the CSF of an adult patient with bacterial meningitis, and strain A42174 was isolated from the blood of an adult with septicemia and no clinical diagnosis of meningitis. Isolates were collected at the Queen Elizabeth Central Hospital (Blantyre, Malawi). Both strains were subjected to whole-genome sequencing using the Illumina genome analyzer GAII platform, as previously described (12). An *in silico* serotyping method was used to confirm the sequenced strains were serotype 1 (67).

The D39 serotype 2 (NCTC 7466) strain was used as a control. A penicillin-resistant serotype 19F ST177 European strain and a tetracycline-resistant serotype 6B ST9533 African strain were used for coinfection experiments. Bacteria were grown overnight at 37°C on blood agar plates containing 5% Horse Blood (Oxoid, Basingstoke, UK) and confirmed to be pneumococcus by detection of α-hemolysis and sensitivity to optochin (Oxoid). Frozen stocks of the bacteria were made by growing a single colony in 20% brain heart infusion (BHI) serum for 8 to 12 h at 37°C before storage at −80°C. These stocks were thawed at room temperature shortly before being required for an experiment.

### Invasive pneumonia model.

Mice were mildly anaesthetized with a mixture of O_2_ and isoflurane (Abbott, Chicago, IL) and infected intra-nasally with 50 μl of PBS containing 10^6^ bacteria, as described previously (68). The mice were scored for signs of disease for the duration of the experiment, according to the scheme of Morton (69) and culled either at predetermined time points or when they became lethargic. The mice were humanely culled by CO_2_ inhalation, followed by cervical dislocation. Blood was obtained by tail bleeds or by cardiac puncture after cervical dislocation to determine the presence of pneumococci at predetermined time points. Lung, brain, and nasopharynx samples were collected and disrupted using a tissue homogenizer (IKA), and bacterial counts were performed using the Miles and Misra method (70).

### Nasopharyngeal carriage model.

Mice were mildly anaesthetized with a mixture of O_2_ and isoflurane and infected intranasally with 10 μl of PBS containing 10^5^ bacteria, as previously described (21).

For the reinfection model of nasopharyngeal carriage, mice were intranasally infected with 10 μl of sterile PBS containing 10^5^ CFU of the bacteremia ST217 serotype 1 strain (A42174) and then reinfected 21 days later with the same dose.

The nasopharyngeal coinfection model was done by using the same dose described; however, once carriage was established, a second infection was performed 7 days postinfection using 10 μl of PBS containing 10^5^ bacteria, but this time of a different serotype. At predetermined time points, mice were humanely culled by CO_2_ inhalation, followed by cervical dislocation. Nasopharyngeal tissue was disrupted using a tissue homogenizer (IKA), and the viable bacterial concentration was determined using the Miles and Misra method (70). The supernatant of homogenized nasopharynx was used for determination of cytokine levels.

### Cytokine analysis.

The levels of nasopharyngeal cytokines and serum IgG of infected mice were determined using mouse ELISA kits (Sigma), with the exception of IL-17, which was performed using an enzyme-linked immunosorbent assay (ELISA) kit from eBiosciences. All ELISAs were performed according to the instructions of the manufacturer.

### RNA extraction.

Growth curves of the serotype 2 (D39) strain and the serotype 1 ST217 bacteremia strain (A42174) were performed by inoculating 250 ml of BHI with 2.5 × 10^6^ bacteria and incubating them at 37°C in a water bath. Samples were taken every hour to determine the optical density at 500 nm (OD_500_). This experiment was performed in triplicate, and the OD_500_ values were plotted to determine three growth phases: the early exponential phase (EEP), the mid-exponential phase (MEP), and the late exponential phase (LEP). RNA from each phase was stabilized by incubating the bacteria in a 5% phenol (Invitrogen) and 95% ethanol solution for 30 min on ice. After stabilization, the RNA was extracted using a phase separation method using TRIzol (Invitrogen) (71). The quantity and quality of RNA was determined using the Qubit fluorometer (Thermo Scientific) and a 2100 Bioanalyzer (Agilent Technologies, Santa Clara, CA), respectively. Illumina NextSeq 500 sequencing (Illumina) was done by Vertis Biotechnologie AG (Freising, Germany).

### Transcriptome analysis.

The quality of the trimmed single-paired-end raw reads from RNA sequencing (RNA-seq) generated using the Illumina platform was checked using FastQC v0.11.5 (www.bioinformatics.babraham.ac.uk/projects/fastqc/). Complete annotated reference genomes for the D39 (accession number NC_008533) and serotype 1 P1031 (accession number CP000920) strains were obtained from GenBank (72) and used for mapping RNA-Seq reads. We then mapped sequence reads for the D39 and serotype 1 (A42174) strains against the D39 and P1031 reference genomes, respectively, for the sequenced RNA samples collected at EEP, MEP, and LEP of *in vitro* growth cultures using bowtie2 v2.3.0 with the default options (73). We converted the annotated reference genome for the D39 and P1031 strains from GenBank to the GFF3 format using the bp_genbank2gff3.pl script (https://metacpan.org/pod/bp_genbank2gff3.pl) and from GFF3 to GTF format using the gff2gtf.pl script (https://github.com/dgpinheiro/bioinfoutilities/blob/master/gff2gtf.pl). To quantify the number of transcripts, we counted the total mapped primary transcripts excluding multi-mapping and multi-overlapping reads for each coding sequence (CDS) in the generated GTF files separately for D39 and serotype 1 strain using featureCounts v2.0.0 (74). Since the quantity of RNA and total number of reads per sample were not necessarily the same, we transformed the raw transcript counts for each strain separately to transcripts per kilobase million (TPM) in R software (https://www.R-project.org/). Since the RNA-seq reads were mapped to different reference genomes, we identified orthologs by clustering all the CDS sequences in D39 and serotype 1 P1031 reference genomes using Panaroo v1.2.3 (75). Using orthologous gene clustering information, CDS sequences found in both genomes (excluding clusters with paralogs) were selected for downstream differential gene analysis (see Table S2 and Fig. S1 in the supplemental material). The logarithms to base 2 of the fold change in gene expression values, i.e., the TPM for serotype 1 divided by D39, were calculated for the genes found in both strains. Heatmaps for visualizing the relative gene expression between serotype 1 A42174 and D39 strains were generated using ComplexHeatmap v2.5.4 in R (76). Comparison of the relative expression patterns of genes according to their functional annotation groups was done using EggNOG-mapper v2.0 (77). Box and dot plots showing the relative gene expression according to functional annotation groups was generated using ggplot2 in R (78).

### Bacterial survival in whole human blood.

The ability of the serotype 2 strain D39 and the serotype 1 ST217 bacteremia strain (A42174) to survive in blood was tested by incubating 10^5^ bacteria in 300 μl of whole human blood from healthy donors. The bacterium-blood mixture was incubated at room temperature in a 360° tube rotator for 24 h. The concentration of viable bacteria was determined using the Miles and Misra method at 0, 1, 2, 4, 6, and 24 h postincubation (70).

### Complement deposition.

Human C3 deposition on the bacterial surface was determined by incubating the bacteria with 20% pooled human serum. The quantification of C3 deposition was quantified by flow cytometry after incubation with a mouse-anti-human C3 antibody (Abcam) and an anti-mouse-APC antibody (eBioscience). To label bacteria, thiazole orange dye (Sigma, Gillingham, UK) was used at a 1:100 dilution. The samples were acquired using a BD Accuri C6 flow cytometer, and C3 deposition was determined using the fluorescent intensity.

### Opsonophagocytosis killing assays.

The human neutrophil cell line HL60 (ATCC CCL-240) was used to perform opsonophagocytosis killing assays according to a modified version of a previously described protocol (79, 80). The pneumococcal isolates were opsonized using pooled human IgG (IVIG; Grifols Therapeutics, New York, NY). After opsonization shaking for 30 min at 37°C, the bacteria were incubated with differentiated HL60 and baby rabbit complement for 45 min at 37°C with shaking. At the end of the assay, the viable bacterial number was determined using the Miles and Misra method (70).

### Statistics.

The statistical analysis was carried out using the GraphPad Prism version 5 statistical package (GraphPad Software, Inc.). A log-rank (Mantel-Cox) test was used to analyze the differences in survival time of infected mice. The remaining *in vivo* experiments were analyzed using either a one-way or a two-way ANOVA, as detailed in the figure legends. The *in vitro* experiments were analyzed by using either a one-way ANOVA or a Student *t* test. Statistical significance is indicated in the figures according to the *P* values (*, *P* < 0.05; **, *P* < 0.01; ***, *P* < 0.005; ****, *P* < 0.001).

### Data availability.

RNA-seq-derived transcriptomic data generated and analyzed in this study have been deposited in the Gene Expression Omnibus (GEO) database under accession number GSE159305.

## References

[1] O’Brien KL, Wolfson LJ, Watt JP, Henkle E, Deloria-Knoll M, McCall N, Lee E, Mulholland K, Levine OS, Cherian T, Hiband Pneumococcal Gloal Burden of Disease Study Team. 2009. Burden of disease caused by *Streptococcus pneumoniae* in children younger than 5 years: global estimates. Lancet 374:893–902. doi:10.1016/S0140-6736(09)61204-6. 19748398

[2] Advisory Committee on Immunization Practices. 1997. Prevention of pneumococcal disease: recommendations of the Advisory Committee on Immunization Practices (ACIP). MMWR Morb Mortal Wkly Rep 46:1–24. 9132580

[3] Bentley SD, Aanensen DM, Mavroidi A, Saunders D, Rabbinowitsch E, Collins M, Donohoe K, Harris D, Murphy L, Quail MA, Samuel G, Skovsted IC, Kaltoft MS, Barrell B, Reeves PR, Parkhill J, Spratt B. 2006. Genetic analysis of the capsular biosynthetic locus from all 90 pneumococcal serotypes. PLoS Genet 2:e31. doi:10.1371/journal.pgen.0020031. 16532061 PMC1391919

[4] Hathaway LJ, Brugger SD, Morand B, Bangert M, Rotzetter JU, Hauser C, Graber WA, Gore S, Kadioglu A, Mühlemann K. 2012. Capsule type of *Streptococcus pneumoniae* determines growth phenotype. PLoS Pathog 8:e1002574. doi:10.1371/journal.ppat.1002574. 22412375 PMC3297593

[5] Abeyta M, Hardy GG, Yother Y. 2003. Genetic alteration of capsule type but not PspA type affects accessibility of surface-bound complement and surface antigens of *Streptococcus pneumoniae*. Infect Immun 71:218–225. doi:10.1128/iai.71.1.218-225.2003. 12496169 PMC143148

[6] Kadioglu A, Weiser JN, Paton JC, Andrew PW. 2008. The role of *Streptococcus pneumoniae* virulence factors in host respiratory colonization and disease. Nat Rev Microbiol 6:288–301. doi:10.1038/nrmicro1871. 18340341

[7] Regev-Yochay G, Raz M, Dagan R, Porat N, Shainberg B, Pinco E, Keller N, Rubinstein E. 2004. Nasopharyngeal carriage of *Streptococcus pneumoniae* by adults and children in community and family settings. Clin Infect Dis 38:632–639. doi:10.1086/381547. 14986245

[8] Gray BM, Converse GM, III, Dillon HC, Jr. 1980. Epidemiologic studies of *Streptococcus pneumoniae* in infants: acquisition, carriage, and infection during the first 24 months of life. J Infect Dis 142:923–933. doi:10.1093/infdis/142.6.923. 7462701

[9] Abdullahi O, Karani A, Tigoi CC, Mugo D, Kungu S, Wanjiru E, Jomo J, Musyimi R, Lipsitch M, Scott JA. 2012. The prevalence and risk factors for pneumococcal colonization of the nasopharynx among children in Kilifi District, Kenya. PLoS One 7:e30787. doi:10.1371/journal.pone.0030787. 22363489 PMC3282706

[10] Ritchie ND, Mitchell TJ, Evans TJ. 2012. What is different about serotype 1 pneumococci? Future Microbiol 7:33–46. doi:10.2217/fmb.11.146. 22191445

[11] Johnson HL, Deloria-Knoll M, Levine OS, Stoszek SK, Freimanis Hance L, Reithinger R, Muenz LR, O’Brien KL. 2010. Systematic evaluation of serotypes causing invasive pneumococcal disease among children under five: the pneumococcal global serotype project. PLoS Med 7:e1000348. doi:10.1371/journal.pmed.1000348. 20957191 PMC2950132

[12] Cornick JE, Chaguza C, Harris SR, Yalcin F, Senghore M, Kiran AM, Govindpershad S, Ousmane S, Plessis M, Pluschke G, Ebruke C, McGee L, Sigaùque B, Collard JM, Antonio M, Gottberg A, French N, Klugman KP, Heyderman RS, Bentley SD, Everett DB, for the PAGE Consortium. 2015. Region-specific diversification of the highly virulent serotype 1 *Streptococcus pneumoniae*. Microbial Genomics 1:e000027. doi:10.1099/mgen.0.000027. 28348812 PMC5320570

[13] Brueggemann AB, Spratt BG. 2003. Geographic distribution and clonal diversity of *Streptococcus pneumoniae* serotype 1 isolates. J Clin Microbiol 41:4966–4970. doi:10.1128/jcm.41.11.4966-4970.2003. 14605125 PMC262517

[14] Cornick JE, Everett DB, Broughton C, Denis BB, Banda DL, Carrol ED, Parry CM. 2011. Invasive *Streptococcus pneumoniae* in children, Malawi, 2004–2006. Emerg Infect Dis 17:1107–1109. doi:10.3201/eid/1706.101404. 21749782 PMC3358202

[15] Everett DB, Cornick J, Denis B, Chewapreecha C, Croucher N, Harris S, Parkhill J, Gordon S, Carrol ED, French N, Heyderman RS, Bentley SD. 2012. Genetic characterization of Malawian pneumococci prior to the roll-out of the PCV13 vaccine using a high-throughput whole-genome sequencing approach. PLoS One 7:e44250. doi:10.1371/journal.pone.0044250. 22970189 PMC3438182

[16] Lourenço J, Obolski U, Swarthout TD, Gori A, Bar-Zeev N, Everett D, Kamng’ona AW, Mwalukomo TS, Mataya AA, Mwansambo C, Banda M, Gupta S, French N, Heyderman RS. 2019. Determinants of high residual post-PCV13 pneumococcal vaccine-type carriage in Blantyre, Malawi: a modeling study. BMC Med 17:210. doi:10.1186/s12916-019-1450-2. 31801542 PMC6894346

[17] Dagan R, Gradstein S, Belmaker I, Porat N, Siton Y, Weber G, Janco J, Yagupsky P. 2000. An outbreak of *Streptococcus pneumoniae* serotype 1 in a closed community in southern Israel. Clin Infect Dis 30:319–321. doi:10.1086/313645. 10671335

[18] Sandgren A, Albiger B, Orihuela CJ, Tuomanen E, Normark S, Henriques-Normark B. 2005. Virulence in mice of pneumococcal clonal types with known invasive disease potential in humans. J Infect Dis 192:791–800. doi:10.1086/432513. 16088828

[19] Syk A, Norman M, Fernebro J, Gallotta M, Farmand S, Sandgren A, Normark S, Henriques-Normark B. 2014. Emergence of hypervirulent mutants resistant to early clearance during systemic serotype 1 pneumococcal infection in mice and humans. J Infect Dis 210:4–13. doi:10.1093/infdis/jiu038. 24443543 PMC4054898

[20] Orihuela CJ, Radin JN, Sublett JE, Gao G, Kaushal D, Tuomanen EI. 2004. Microarray analysis of pneumococcal gene expression during invasive disease. Infect Immun 72:5582–5596. doi:10.1128/IAI.72.10.5582-5596.2004. 15385455 PMC517545

[21] Richards L, Ferreira DM, Miyaji EN, Andrew PW, Kadioglu A. 2010. The immunizing effect of pneumococcal nasopharyngeal colonization: protection against future colonization and fatal invasive disease. Immunobiology 215:251–263. doi:10.1016/j.imbio.2009.12.004. 20071053

[22] Kamng’ona AW, Hinds J, Bar-Zeev N, Gould KA, Chaguza C, Msefula C, Cornick JE, Kulohoma BW, Gray K, Bentley SD, French N, Heyderman RS, Everett DB. 2015. High multiple carriage and emergence of *Streptococcus pneumoniae* vaccine serotype variants in Malawian children. BMC Infect Dis 15:234. doi:10.1186/s12879-015-0980-2. 26088623 PMC4474563

[23] Georgieva M, Kagedan L, Lu Y-J, Thompson CM, Lipsitch M. 2018. Antigenic variation in *Streptococcus pneumoniae* PspC promotes immune escape in the presence of variant-specific immunity. mBio 9:e00264-18. doi:10.1128/mBio.00264-18. 29535198 PMC5850329

[24] Bradshaw JL, Pipkins HL, Keller LE, Pendarvis JK, McDaniel LS. 2018. Mucosal infections and invasive potential of nonencapsulated *Streptococcus pneumoniae* are enhanced by oligopeptide binding proteins AliC and AliD. mBio 9:e02097-17. doi:10.1128/mBio.02097-17. 29339428 PMC5770551

[25] Gómez-Mejia A, Gámez G, Hirschmann S, Kluger V, Rath H, Böhm S, Voss F, Kakar N, Petruschka L, Völker U, Brückner R, Mäder U, Hammerschmidt S. 2018. Pneumococcal metabolic adaptation and colonization are regulated by the two-component regulatory system 08. mSphere 3:e00165-18. doi:10.1128/mSphere.00165-18. 29769380 PMC5956151

[26] Nagaoka K, Yamashita Y, Kimura H, Suzuki M, Konno S, Fukumoto T, Akizawa K, Morinaga Y, Yanagihara K, Nishimura M. 2019. Effects of anaerobic culturing on pathogenicity and virulence-related gene expression in pneumococcal pneumonia. J Infect Dis 219:1545–1553. doi:10.1093/infdis/jiy718. 30561674

[27] Tuomanen EI. 1997. The biology of pneumococcal infection. Pediatr Res 42:253–258. doi:10.1203/00006450-199709000-00001. 9284261

[28] Buckwalter CM, King SJ. 2012. Pneumococcal carbohydrate transport: food for thought. Trends Microbiol 20:517–522. doi:10.1016/j.tim.2012.08.008. 22959614 PMC4630977

[29] Singh AK, Pluvinage B, Higgins MA, Dalia AB, Woodiga SA, Flynn M, Lloyd AR, Weiser JN, Stubbs KA, Boraston AB, King SJ. 2014. Unraveling the multiple functions of the architecturally intricate *Streptococcus pneumoniae* β-galactosidase, BgaA. PLoS Pathog 10:e1004364. doi:10.1371/journal.ppat.1004364. 25210925 PMC4161441

[30] Claverys J-P, Grossiord B, Alloing G. 2000. Is the Ami-AliA/B oligopeptide permease of *Streptococcus pneumoniae* involved in sensing environmental conditions? Res Microbiol 151:457–463. doi:10.1016/s0923-2508(00)00169-8. 10961459

[31] Kerr AR, Adrian PV, Estevão S, de Groot R, Alloing G, Claverys JP, Mitchell TJ, Hermans PW. 2004. The Ami-AliA/AliB permease of *Streptococcus pneumoniae* is involved in nasopharyngeal colonization but not in invasive disease. Infect Immun 72:3902–3906. doi:10.1128/IAI.72.7.3902-3906.2004. 15213133 PMC427416

[32] Bidossi A, Mulas L, Decorosi F, Colomba L, Ricci S, Pozzi G, Deutscher J, Viti C, Oggioni MR. 2012. A functional genomics approach to establish the complement of carbohydrate transporters in *Streptococcus pneumoniae*. PLoS One 7:e33320. doi:10.1371/journal.pone.0033320. 22428019 PMC3302838

[33] Nieto C, Puyet A, Espinosa M. 2001. MalR-mediated regulation of the *Streptococcus pneumoniae malMP* operon at promoter PM: influence of a proximal divergent promoter region and competition between MalR and RNA polymerase proteins. J Biol Chem 276:14946–14954. doi:10.1074/jbc.M010911200. 11278784

[34] Puyet A, Ibanez AM, Espinosa M. 1993. Characterization of the *Streptococcus pneumoniae* maltosaccharide regulator MalR, a member of the Lad-GalR family of repressors displaying distinctive genetic feature. J Biol Chem 268:25402–25408. 8244973

[35] Ogunniyi AD, Mahdi LK, Trappetti C, Verhoeven N, Mermans D, Van der Hoek MB, Plumptre CD, Paton JC. 2012. Identification of genes that contribute to the pathogenesis of invasive pneumococcal disease by *in vivo* transcriptomic analysis. Infect Immun 80:3268–3278. doi:10.1128/IAI.00295-12. 22778095 PMC3418729

[36] Tyx RE, Roche-Hakansson H, Hakansson AP. 2011. Role of dihydrolipoamide dehydrogenase in regulation of raffinose transport in *Streptococcus pneumonia*e. J Bacteriol 193:3512–3524. doi:10.1128/JB.01410-10. 21602335 PMC3133304

[37] Rosenow C, Maniar M, Trias J. 1999. Regulation of the α-galactosidase activity in *Streptococcus pneumoniae*: characterization of the raffinose utilization system. Genome Res 9:1189–1197. doi:10.1101/gr.9.12.1189. 10613841 PMC311000

[38] Dawid S, Roche AM, Weiser JN. 2007. The Blp bacteriocins of *Streptococcus pneumoniae* mediate intraspecies competition both *in vitro* and *in vivo*. Infect Immun 75:443–451. doi:10.1128/IAI.01775-05. 17074857 PMC1828380

[39] van Opijnen T, Camilli A. 2012. A fine scale phenotype-genotype virulence map of a bacterial pathogen. Genome Res 22:2541–2551. doi:10.1101/gr.137430.112. 22826510 PMC3514683

[40] King SJ. 2010. Pneumococcal modification of host sugars: a major contributor to colonization of the human airway? Mol Oral Microbiol 25:15–24. doi:10.1111/j.2041-1014.2009.00564.x. 20331791

[41] Dalia AB, Standish AJ, Weiser JN. 2010. Three surface exoglycosidases from *Streptococcus pneumoniae*, NanA, BgaA, and StrH, promote resistance to opsonophagocytic killing by human neutrophils. Infect Immun 78:2108–2116. doi:10.1128/IAI.01125-09. 20160017 PMC2863504

[42] Limoli DH, Sladek JA, Fuller LA, Singh AK, King SJ. 2011. BgaA acts as an adhesin to mediate attachment of some pneumococcal strains to human epithelial cells. Microbiology (Reading) 157:2369–2381. doi:10.1099/mic.0.045609-0. 21602213 PMC3167885

[43] Jeong JK, Kwon O, Lee YM, Oh DB, Lee JM, Kim S, Kim EH, Le TN, Rhee DK, Kang HA. 2009. Characterization of the *Streptococcus pneumoniae* BgaC protein as a novel surface beta-galactosidase with specific hydrolysis activity for the Galβ1-3GlcNAc moiety of oligosaccharides. J Bacteriol 191:3011–3023. doi:10.1128/JB.01601-08. 19270088 PMC2681812

[44] Perez-Dorado I, Galan-Bartual S, Hermoso JA. 2012. Pneumococcal surface proteins: when the whole is greater than the sum of its parts. Mol Oral Microbiol 27:221–245. doi:10.1111/j.2041-1014.2012.00655.x. 22759309

[45] Asmat TM, Klingbeil K, Jensch I, Burchhardt G, Hammerschmidt S. 2012. Heterologous expression of pneumococcal virulence factor PspC on the surface of *Lactococcus lactis* confers adhesive properties. Microbiology (Reading) 158:771–780. doi:10.1099/mic.0.053603-0. 22222496

[46] Marra A, Lawson S, Asundi JS, Brigham D, Hromockyj AE. 2002. *In vivo* characterization of the *psa* genes from *Streptococcus pneumoniae* in multiple models of infection. Microbiology 148:1483–1491. doi:10.1099/00221287-148-5-1483. 11988523

[47] Bergmann S, Schoenen H, Hammerschmidt S. 2013. The interaction between bacterial enolase and plasminogen promotes adherence of *Streptococcus pneumoniae* to epithelial and endothelial cells. Int J Med Microbiol 303:452–462. doi:10.1016/j.ijmm.2013.06.002. 23906818

[48] Paterson GK, Mitchell TJ. 2006. The role of *Streptococcus pneumoniae* sortase A in colonization and pathogenesis. Microbes Infect 8:145–153. doi:10.1016/j.micinf.2005.06.009. 16182588

[49] Ianelli F, Pearce B, Pozzi G. 1999. The type 2 capsule locus of *Streptococcus pneumoniae*. J Bacteriol 181:2652–2654. doi:10.1128/JB.181.8.2652-2654.1999. 10198036 PMC93698

[50] Mitchell T, Dalziel C. 2014. The biology of pneumolysin, MACPF/CDC proteins: agents of defence, attack, and invasion. Subcel Biochem 80:145–160. doi:10.1007/978-94-017-8881-6_8. 24798011

[51] Amaral FE, Parker D, Randis TM, Kulkarni R, Prince AS, Shirasu-Hiza MM, Ratner AJ. 2015. Rational manipulation of mRNA folding free energy allows rheostat control of pneumolysin production by *Streptococcus pneumoniae*. PLoS One 10:e0119823. doi:10.1371/journal.pone.0119823. 25798590 PMC4370707

[52] Walker SG, Carnu OI, Tuter G, Ryan ME. 2006. The immunoglobulin A1 proteinase from *Streptococcus pneumoniae* is inhibited by tetracycline compounds. FEMS Immunol Med Microbiol 48:218–222. doi:10.1111/j.1574-695X.2006.00148.x. 16995879

[53] Kadioglu A, Brewin H, Härtel T, Brittan JL, Klein M, Hammerschmidt S, Jenkinson HF. 2010. Pneumococcal protein PavA is important for nasopharyngeal carriage and development of sepsis. Mol Oral Microbiol 25:50–60. doi:10.1111/j.2041-1014.2009.00561.x. 20331793

[54] Pracht D, Elm C, Gerber J, Bergmann S, Rohde M, Seiler M, Kim KS, Jenkinson HF, Nau R, Hammerschmidt S. 2005. PavA of *Streptococcus pneumoniae* modulates adherence, invasion, and meningeal inflammation. Infect Immun 73:2680–2689. doi:10.1128/IAI.73.5.2680-2689.2005. 15845469 PMC1087317

[55] Feldman C, Cockeran R, Jedrzejas MJ, Mitchell TJ, Anderson R. 2007. Hyaluronidase augments pneumolysin-mediated injury to human ciliated epithelium. Int J Infect Dis 11:11–15. doi:10.1016/j.ijid.2005.09.002. 16483814

[56] Mehr S, Wood N. 2012. *Streptococcus pneumoniae*: a review of carriage, infection, serotype replacement, and vaccination. Paediatr Respir Rev 13:258–264. doi:10.1016/j.prrv.2011.12.001. 23069126

[57] Bogaert D, de Groot R, Hermans P. 2004. *Streptococcus pneumoniae* colonization: the key to pneumococcal disease. Lancet Infect Dis 4:144–154. doi:10.1016/S1473-3099(04)00938-7. 14998500

[58] Neill DR, Coward WR, Gritzfeld JF, Richards L, Garcia-Garcia FJ, Dotor J, Gordon SB, Kadioglu A. 2014. Density and duration of pneumococcal carriage is maintained by transforming growth factor β1 and T regulatory cells. Am J Respir Crit Care Med 189:1250–1259. doi:10.1164/rccm.201401-0128OC. 24749506 PMC4225851

[59] Bordon J, Aliberti S, Fernandez-Botran R, Uriarte SM, Rane MJ, Duvvuri P, Peyrani P, Morlacchi LC, Blasi F, Ramirez JA. 2013. Understanding the roles of cytokines and neutrophil activity and neutrophil apoptosis in the protective versus deleterious inflammatory response in pneumonia. Int J Infect Dis 17:e76–e83. doi:10.1016/j.ijid.2012.06.006. 23069683

[60] Minhas V, Harvey RM, McAllister LJ, Seemann T, Syme AE, Baines SL, Paton JC, Trappetti C. 2019. Capacity to utilize raffinose dictates pneumococcal disease phenotype. mBio 10:e02596. doi:10.1128/mBio.02596-18. 30647157 PMC6336424

[61] Hyams C, Trzcinski K, Camberlein E, Weinberger DM, Chimalapati S, Noursadeghi M, Lipsitch M, Brown JS. 2013. *Streptococcus pneumoniae* capsular serotype invasiveness correlates with the degree of factor H binding and opsonization with C3b/iC3b. Infect Immun 81:354–363. doi:10.1128/IAI.00862-12. 23147038 PMC3536142

[62] Kjos M, Miller E, Slager J, Lake FB, Gericke O, Roberts IS, Rozen DE, Veening JW. 2016. Expression of *Streptococcus pneumoniae* bacteriocins is induced by antibiotics via regulatory interplay with the competence system. PLoS Pathog 12:e1005422. doi:10.1371/journal.ppat.1005422. 26840404 PMC4739728

[63] Austrian R, Gold J. 1964. Pneumococcal bacteremia with especial reference to bacteremic pneumococcal pneumonia. Ann Intern Med 60:759–776. doi:10.7326/0003-4819-60-5-759. 14156606

[64] Harboe ZB, Thomsen RW, Riis A, Valentiner-Branth P, Christensen JJ, Lambertsen L, Krogfelt KA, Konradsen HB, Benfield TL. 2009. Pneumococcal serotypes and mortality following invasive pneumococcal disease: a population-based cohort study. PLoS Med 6:E1000081. doi:10.1371/journal.pmed.1000081. 19468297 PMC2680036

[65] Feldman C, Anderson R. 2014. Review: current and new generation pneumococcal vaccines. J Infect 69:309–325. doi:10.1016/j.jinf.2014.06.006. 24968238

[66] Grogg SE, Schultz J. 2015. Call to action on pneumococcal disease: review of vaccination evidence and outcomes of webcast programs. J Am Osteopath Assoc 115:S6–25. doi:10.7556/jaoa.2015.071. 26000904

[67] Croucher NJ, Harris SR, Fraser C, Quail MA, Burton J, van der Linden M, McGee L, von Gottberg A, Song JH, Ko KS, Pichon B, Baker S, Parry CM, Lambertsen LM, Shahinas D, Pillai DR, Mitchell TJ, Dougan G, Tomasz A, Klugman KP, Parkhill J, Hanage WP, Bentley SD. 2011. Rapid pneumococcal evolution in response to clinical interventions. Science 331:430–434. doi:10.1126/science.1198545. 21273480 PMC3648787

[68] Kadioglu A, Gingles NA, Grattan K, Kerr A, Mitchell TJ, Andrew PW. 2000. Host cellular immune response to pneumococcal lung infection in mice. Infect Immun 68:492–501. doi:10.1128/iai.68.2.492-501.2000. 10639409 PMC97168

[69] Morton DB, Griffiths PH. 1985. Guidelines on the recognition of pain, distress, and discomfort in experimental animals and an hypothesis for assessment. Vet Rec 116:431–436. doi:10.1136/vr.116.16.431. 3923690

[70] Miles AA, Misra SS, Irwin JO. 1938. The estimation of the bactericidal power of the blood. J Hyg (Lond) 38:732–749. doi:10.1017/s002217240001158x. PMC2199673 20475467

[71] Kroger C, Dillon SC, Cameron ADS, Papenfort K, Sivasankaran SK, Hokamp K, Chao Y, Sittka A, Hebrard M, Handler K, Colgan A, Leekitcharoenphon P, Langridge GC, Lohan AJ, Loftus B, Lucchini S, Ussery DW, Dorman CJ, Thomson NR, Vogel J, Hinton JCD. 2012. The transcriptional landscape and small RNAs of *Salmonella enterica* serovar Typhimurium. Proc Natl Acad Sci U S A 109:E1277–E1286. doi:10.1073/pnas.1201061109. 22538806 PMC3356629

[72] Clark K, Karsch-Mizrachi I, Lipman DJ, Ostell J, Sayers EW. 2016. GenBank. Nucleic Acids Res 44:D67–D72. doi:10.1093/nar/gkv1276. 26590407 PMC4702903

[73] Langmead B, Salzberg SL. 2012. Fast gapped-read alignment with Bowtie 2. Nat Methods 9:357–359. doi:10.1038/nmeth.1923. 22388286 PMC3322381

[74] Liao Y, Smyth GK, Shi W. 2014. featureCounts: an efficient general purpose program for assigning sequence reads to genomic features. Bioinformatics 30:923–930. doi:10.1093/bioinformatics/btt656. 24227677

[75] Tonkin-Hill G, MacAlasdair N, Ruis C, Weimann A, Horesh G, Lees JA, Gladstone RA, Lo S, Beaudoin C, Floto RA, Frost SDW, Corander J, Bentley SD, Parkhill J. 2020. Producing polished prokaryotic pangenomes with the Panaroo pipeline. Genome Biol 21:180. doi:10.1186/s13059-020-02090-4. 32698896 PMC7376924

[76] Gu Z, Eils R, Schlesner M. 2016. Complex heatmaps reveal patterns and correlations in multidimensional genomic data. Bioinformatics 32:2847–2849. doi:10.1093/bioinformatics/btw313. 27207943

[77] Huerta-Cepas J, Szklarczyk D, Heller D, Hernández-Plaza A, Forslund SK, Cook H, Mende DR, Letunic I, Rattei T, Jensen LJ, von Mering C, Bork P. 2019. EggNOG 5.0: a hierarchical, functionally and phylogenetically annotated orthology resource based on 5090 organisms and 2502 viruses. Nucleic Acids Res 47:D309–D314. doi:10.1093/nar/gky1085. 30418610 PMC6324079

[78] Wickham H. 2016. Ggplot2: elegant graphics for data analysis. Springer International Publishing, New York, NY. ISBN 978-3-319-24277-4.

[79] Romero-Steiner S, Frasch C, Concepcion N, Goldblatt D, Kayhty H, Vakevainen M, Laferriere C, Wauters D, Nahm MH, Schinsky MF, Plikaytis BD, Carlone GM. 2003. Multilaboratory evaluation of a viability assay for measurement of opsonophagocytic antibodies specific to the capsular polysaccharides of *Streptococcus pneumoniae*. Clin Diagn Lab Immunol 10:1019–1024. doi:10.1128/cdli.10.6.1019-1024.2003. 14607861 PMC262452

[80] Bangert M, Bricio-Moreno L, Gore S, Rajam G, Ades EW, Gordon SB, Kadioglu A. 2012. P4-mediated antibody therapy in an acute model of invasive pneumococcal disease. J Infect Dis 205:1399–1407. doi:10.1093/infdis/jis223. 22457294

