# Supplementary figures and images for: Lower Density and Shorter Duration of Nasopharyngeal Carriage by Pneumococcal Serotype 1 (ST217) May Explain Its Increased Invasiveness over Other Serotypes
Source: mBio. 2020 Dec 8;11(6):e00814-20. doi: 10.1128/mBio.00814-20 (PMC7733939; doi:10.1128/mBio.00814-20)

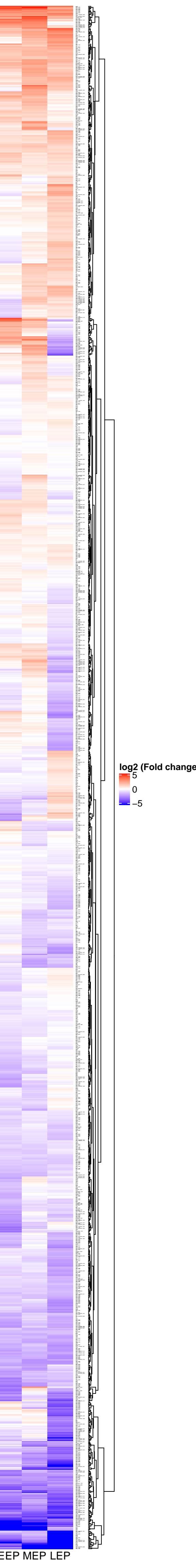

Supplement: FIG S1 [file mBio.00814-20-sf001.pdf]

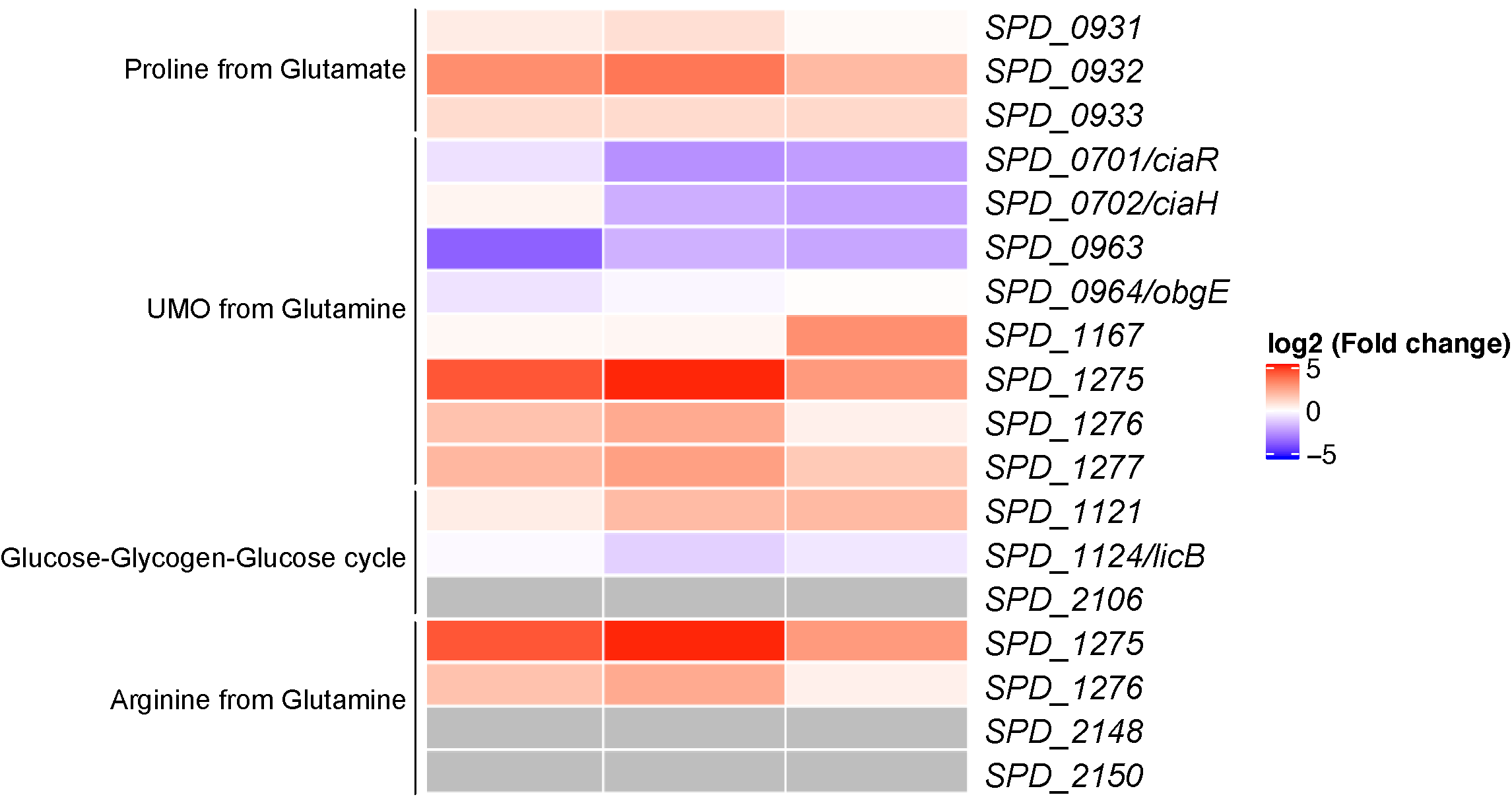

Supplement: FIG S2 [file mBio.00814-20-sf002.tif]
